# 9α-Hy­droxy-12-{[4-(4-hy­droxy­phen­yl)piperazin-1-yl]meth­yl}-4,8-dimethyl-3,14-dioxatri­cyclo­[9.3.0.0^2,4^]tetra­dec-7-en-13-one

**DOI:** 10.1107/S1600536814007430

**Published:** 2014-04-09

**Authors:** Mohamed Loubidi, Ahmed Benharref, Lahcen El Ammari, Mohamed Saadi, Moha Berraho

**Affiliations:** a Laboratoire de Chimie Biomoleculaire, Substances Naturelles et Réactivité URAC16, Faculté des Sciences Semlalia, BP 2390 Bd My Abdellah, 40000 Marrakech, Morocco; b Laboratoire de Chimie du Solide Appliquée, Faculté des Sciences, Université Mohammed V-Agdal, BP 1014, Avenue Ibn Battouta, Rabat, Morocco

## Abstract

The title compound, C_25_H_34_N_2_O_5_, was synthesized from 9α-hy­droxy­parthenolide (9α-hy­droxy-4,8-dimethyl-12-methylen-3, 14-dioxa-tri­cyclo­[9.3.0.0^2,4^]tetra­dec-7-en-13-one), which in turn was isolated from the chloro­form extract of the aerial parts of *Anvillea radiata*. The mol­ecule comprises a ten-membered ring fused to a five-membered ring with an additional ep­oxy ring system fused to the ten-membered ring. The five-membered ring also carries a 4-hy­droxy­phenyl-piperazin-1-ylmethyl substituent. The ten-membered ring adopts an approximate chair–chair conformation, while the piperazine ring displays a chair conformation and the five-membered ring shows an envelope conformation with the C atom closest to the hy­droxy group forming the flap. Two C atoms in the phenyl ring and the O atom of the hydroxyl group are disordered over two sites, with an occupancy ratio of 0.53 (5):0.47 (5). An intra­molecular O—H⋯N hydrogen-bond stabilizes the mol­ecular conformation. In the crystal, C—H⋯O hydrogen bonds link the mol­ecules into zigzag chains running along the *a-*axis direction.

## Related literature   

For background to the medicinal uses of the plant *Anvillea radiata*, see: Abdel Sattar *et al.* (1996 ▶); El Hassany *et al.* (2004 ▶). For the reactivity of this sesquiterpene, see: Hwang *et al.* (2006 ▶); Neelakantan *et al.* (2009 ▶). For a related synthetic procedure, see: Moumou *et al.* (2012 ▶). For conformational analysis, see: Cremer & Pople (1975 ▶).
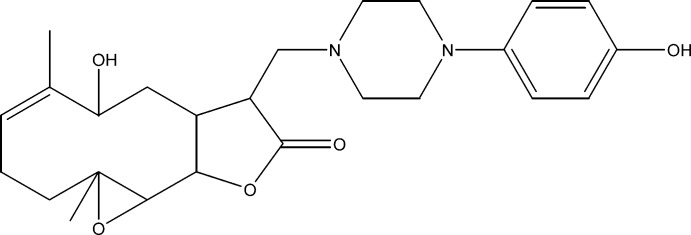


## Experimental   

### 

#### Crystal data  

C_25_H_34_N_2_O_5_
*M* *_r_* = 442.54
Monoclinic, 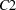

*a* = 29.880 (5) Å
*b* = 6.841 (5) Å
*c* = 11.999 (5) Å
β = 102.307 (5)°
*V* = 2396 (2) Å^3^
*Z* = 4
Mo *K*α radiation
μ = 0.09 mm^−1^
*T* = 296 K
0.5 × 0.03 × 0.03 mm

#### Data collection  

Bruker X8 APEX Diffractometer
Absorption correction: multi-scan (*SADABS*; Bruker, 2009 ▶) *T*_min_ = 0.639, *T*_max_ = 0.747
13097 measured reflections
2868 independent reflections
1830 reflections with *I* > 2σ(*I*)
*R*_int_ = 0.055

#### Refinement  

*R*[*F* ^2^ > 2σ(*F* ^2^)] = 0.044
*wR*(*F* ^2^) = 0.102
*S* = 1.08
2868 reflections
322 parameters
1 restraint
H-atom parameters constrained
Δρ_max_ = 0.14 e Å^−3^
Δρ_min_ = −0.14 e Å^−3^

### 

Data collection: *APEX2* (Bruker, 2009 ▶); cell refinement: *SAINT* (Bruker, 2009 ▶); data reduction: *SAINT*; program(s) used to solve structure: *SHELXS97* (Sheldrick, 2008 ▶); program(s) used to refine structure: *SHELXL97* (Sheldrick, 2008 ▶); molecular graphics: *ORTEP-3 for Windows* (Farrugia, 2012 ▶); software used to prepare material for publication: *PLATON* (Spek, 2009 ▶) and *publCIF* (Westrip, 2010 ▶).

## Supplementary Material

Crystal structure: contains datablock(s) I, global. DOI: 10.1107/S1600536814007430/sj5394sup1.cif

Structure factors: contains datablock(s) I. DOI: 10.1107/S1600536814007430/sj5394Isup2.hkl

Supporting information file. DOI: 10.1107/S1600536814007430/sj5394Isup3.cml

CCDC reference: 995311

Additional supporting information:  crystallographic information; 3D view; checkCIF report

## Figures and Tables

**Table 1:** Hydrogen-bond geometry (Å, °)

*D*—H⋯*A*	*D*—H	H⋯*A*	*D*⋯*A*	*D*—H⋯*A*
O3—H3⋯N1	0.82	2.22	3.030 (4)	169
C2—H2⋯O4^i^	0.98	2.45	3.243 (4)	138
C4—H4*A*⋯O2^ii^	0.97	2.43	3.315 (5)	151

Symmetry codes: (i) 
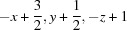
; (ii) 
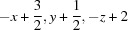
.

**Fig. 1.**
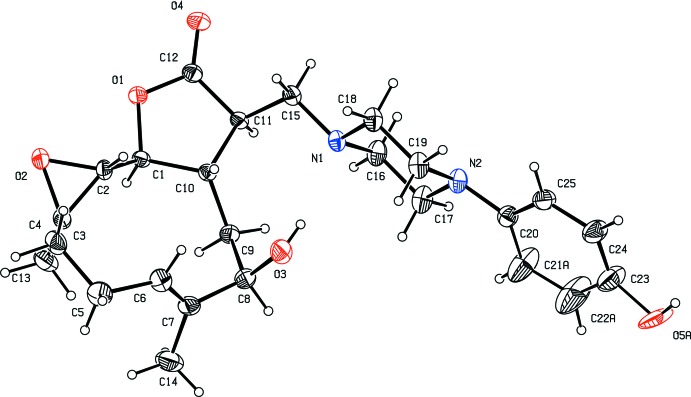
:The molecular structure of the title compound with the atom-labelling scheme. Displacement ellipsoids are drawn at the 30% probability level. H atoms are represented as small spheres of arbitrary radii. For clarity only the major disorder components of the disordered C21 C22 and O5 atoms are shown.

**Fig. 2.**
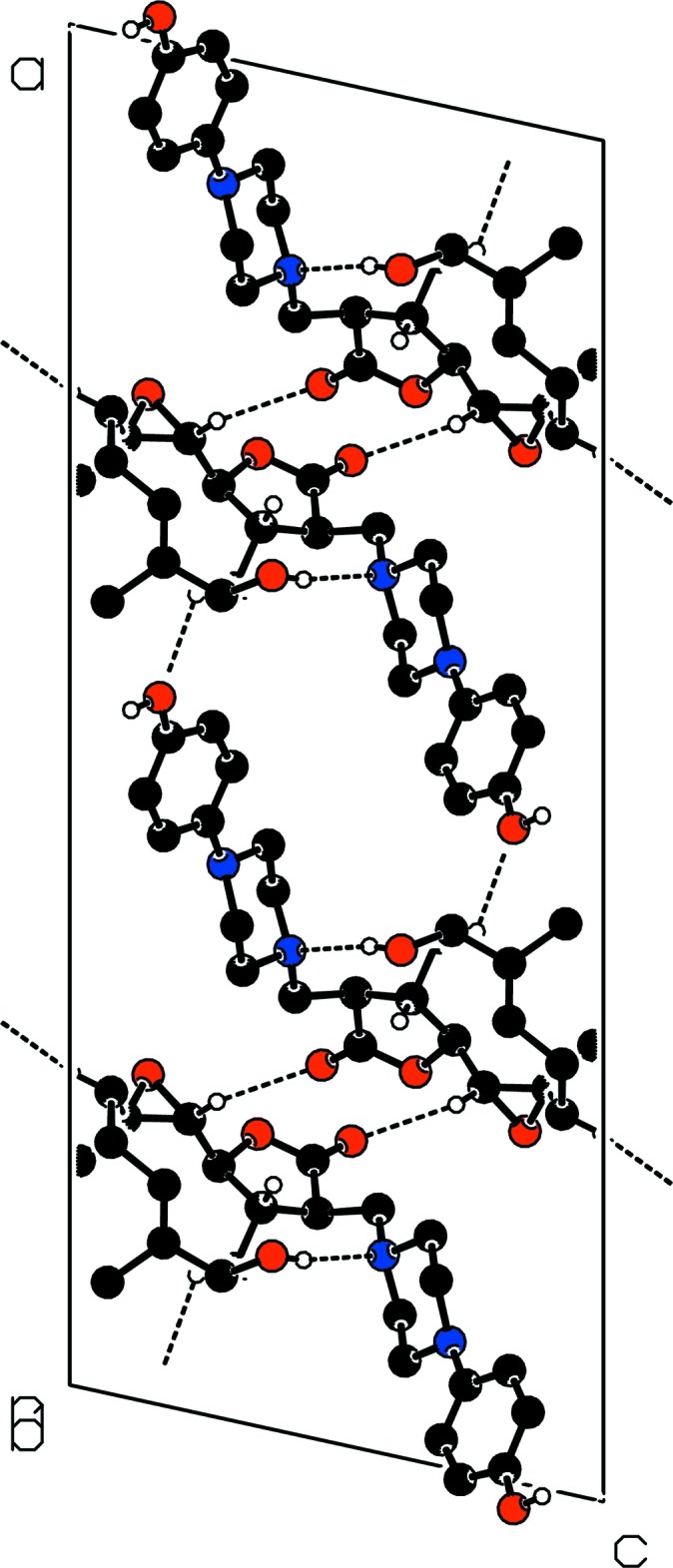
: Packing view showing the C–H···O and O–H···N hydrogen bonds as dashed lines. H atoms not involved in hydrogen bonding and the atoms of the minor disorder component have been omitted for clarity.

## supplementary crystallographic information

### 1. Comment

Our work lies within the framework of the evaluation of medicinal plants and in particular, *Anvillea radiata*. The main constituent of the chloroform extract of the aerial parts of this plant is 9α-hydroxypartenolide (Abdel Sattar *et al.*, 1996; El Hassany *et al.*, 2004). The reactivity of this sesquiterpene lactone and its derivatives has been the subject of several studies (Hwang *et al.*, 2006; Neelakantan *et al.*, 2009; Moumou *et al.*, 2012), in order to prepare high value added products for use in industrial pharmacology. In this work we present the crystal structure of the title compound (I). The molecule is built up from fused five-and ten-membered rings with the hydroxyphenyl-pyperazine group as a substituent. An additional epoxy ring system is also fused to the ten-membered ring at C2 and C3. The molecular structure of (I), Fig.1, shows the lactone ring to adopt an envelope conformation, as indicated by the Cremer & Pople (1975) puckering parameters QT = 0.208 (3) Å and φ2 = 248.2 (7)°. The ten-membered ring displays an approximate chair-chair conformation, while the pyperazine ring has a perfect chair conformation with QT = 0.572 (3) Å, θ = 176.9 (3) and φ2 = 184 (5)°. An intramolecular O4—H4···N1 hydrogen bond is observed. In the crystal, C—H···O hydrogen bonds link the molecules into zigzag chains running along the *a* axis (Table 1, Fig 1).

### 2. Experimental

A mixture of 9α-hydoxypartenolide (9α-hydroxy-4,8-dimethyl-12-methylene- 3,14-dioxatricyclo[9.3.0.0^2^,^4^]tetradec-7-en-13-one) (1 g, 3.8 mmol) and one equivalent of 1-(4-hydroxyphenyl-piperazine) in EtOH (20 ml) was stirred for twelve hours at room temperature. The reaction was stopped by adding water (10 ml) and the solution extracted with chloroform (3 *x* 20 ml). The combined organic layers were dried over anhydrous MgSO_4_, filtered and concentrated under vacuum to give 1.5 g (3.3 mmol) of the title compound (yield: 90%). Recrystallization was performed from ethyl acetate.

### 3. Refinement

All H atoms were fixed geometrically and treated as riding with C—H = 0.96 Å (methyl), 0.97 Å (methylene), 0.98 Å (methine) and O–H = 0.82 Å with *U*_iso_(H) = 1.2*U*_eq_ (methylene, methine) or *U*_iso_(H) = 1.5*U*_eq_ (methyl, OH). The O5, C22 and C21 atoms are disordered over two positions. The occupancy factors for these sites were refined and converged to the ratio 0.53 (5):0.47 (5). In the absence of significant anomalous scattering, the absolute configuration could not be reliably determined and any references to the Flack parameter were removed.

### Crystal data

**Table:**

C_25_H_34_N_2_O_5_	*F*(000) = 952
*M**_r_* = 442.54	*D*_x_ = 1.227 Mg m^−^^3^
Monoclinic, *C*2	Mo *K*α radiation, λ = 0.71073 Å
Hall symbol: C 2y	Cell parameters from 4801 reflections
*a* = 29.880 (5) Å	θ = 2.5–27.1°
*b* = 6.841 (5) Å	µ = 0.09 mm^−^^1^
*c* = 11.999 (5) Å	*T* = 296 K
β = 102.307 (5)°	Needle, colourless
*V* = 2396 (2) Å^3^	0.5 × 0.03 × 0.03 mm
*Z* = 4	

### Data collection

**Table:**

Bruker X8 APEX Diffractometer	2868 independent reflections
Radiation source: fine-focus sealed tube	1830 reflections with *I* > 2σ(*I*)
Graphite monochromator	*R*_int_ = 0.055
φ and ω scans	θ_max_ = 27.1°, θ_min_ = 2.5°
Absorption correction: multi-scan (*SADABS*; Bruker, 2009)	*h* = −38→38
*T*_min_ = 0.639, *T*_max_ = 0.747	*k* = −8→7
13097 measured reflections	*l* = −15→15

### Refinement

**Table:**

Refinement on *F*^2^	Secondary atom site location: difference Fourier map
Least-squares matrix: full	Hydrogen site location: inferred from neighbouring sites
*R*[*F*^2^ > 2σ(*F*^2^)] = 0.044	H-atom parameters constrained
*wR*(*F*^2^) = 0.102	*w* = 1/[σ^2^(*F*_o_^2^) + (0.0408*P*)^2^] where *P* = (*F*_o_^2^ + 2*F*_c_^2^)/3
*S* = 1.08	(Δ/σ)_max_ = 0.001
2868 reflections	Δρ_max_ = 0.14 e Å^−^^3^
322 parameters	Δρ_min_ = −0.14 e Å^−^^3^
1 restraint	Extinction correction: *SHELXL*, Fc^*^=kFc[1+0.001xFc^2^λ^3^/sin(2θ)]^-1/4^
Primary atom site location: structure-invariant direct methods	Extinction coefficient: 0.0063 (6)

### Special details

**Table:**

Geometry. All s.u.'s (except the s.u. in the dihedral angle between two l.s. planes) are estimated using the full covariance matrix. The cell s.u.'s are taken into account individually in the estimation of s.u.'s in distances, angles and torsion angles; correlations between s.u.'s in cell parameters are only used when they are defined by crystal symmetry. An approximate (isotropic) treatment of cell s.u.'s is used for estimating s.u.'s involving l.s. planes.
Refinement. Refinement of *F*^2^ against ALL reflections. The weighted *R*-factor *wR* and goodness of fit *S* are based on *F*^2^, conventional *R*-factors *R* are based on *F*, with *F* set to zero for negative *F*^2^. The threshold expression of *F*^2^ > 2σ(*F*^2^) is used only for calculating *R*-factors(gt) *etc*. and is not relevant to the choice of reflections for refinement. *R*-factors based on *F*^2^ are statistically about twice as large as those based on *F*, and *R*- factors based on ALL data will be even larger.

### Fractional atomic coordinates and isotropic or equivalent isotropic displacement parameters (Å^2^)

**Table:**

	*x*	*y*	*z*	*U*_iso_*/*U*_eq_	Occ. (<1)
C1	0.81526 (10)	0.5109 (5)	0.7206 (2)	0.0388 (7)	
H1	0.8380	0.4421	0.7782	0.047*	
C2	0.78537 (10)	0.6359 (5)	0.7759 (2)	0.0425 (7)	
H2	0.7681	0.7338	0.7245	0.051*	
C3	0.79457 (11)	0.6940 (5)	0.8964 (2)	0.0478 (8)	
C4	0.77809 (13)	0.8925 (6)	0.9221 (3)	0.0645 (10)	
H4A	0.7699	0.8898	0.9961	0.077*	
H4B	0.7508	0.9254	0.8655	0.077*	
C5	0.81482 (14)	1.0505 (5)	0.9224 (3)	0.0687 (11)	
H5A	0.8007	1.1787	0.9185	0.082*	
H5B	0.8376	1.0428	0.9931	0.082*	
C6	0.83780 (13)	1.0258 (5)	0.8232 (3)	0.0548 (9)	
H6	0.8195	1.0503	0.7516	0.066*	
C7	0.88024 (13)	0.9743 (5)	0.8241 (3)	0.0544 (9)	
C8	0.89540 (12)	0.9174 (5)	0.7157 (3)	0.0545 (9)	
H8	0.9283	0.9432	0.7273	0.065*	
C9	0.88783 (10)	0.6976 (5)	0.6916 (2)	0.0464 (8)	
H9A	0.9070	0.6580	0.6397	0.056*	
H9B	0.8985	0.6275	0.7625	0.056*	
C10	0.83879 (10)	0.6316 (4)	0.6409 (2)	0.0369 (7)	
H10	0.8203	0.7490	0.6184	0.044*	
C11	0.83398 (10)	0.5009 (5)	0.5360 (2)	0.0420 (7)	
H11	0.8624	0.4278	0.5393	0.050*	
C12	0.79599 (11)	0.3623 (5)	0.5444 (2)	0.0452 (8)	
C13	0.83375 (13)	0.6105 (6)	0.9824 (2)	0.0674 (11)	
H13A	0.8596	0.6970	0.9913	0.101*	
H13B	0.8419	0.4852	0.9566	0.101*	
H13C	0.8249	0.5957	1.0542	0.101*	
C14	0.91847 (14)	0.9532 (7)	0.9290 (3)	0.0855 (13)	
H14A	0.9427	1.0427	0.9241	0.128*	
H14B	0.9300	0.8218	0.9334	0.128*	
H14C	0.9069	0.9817	0.9959	0.128*	
C15	0.82137 (11)	0.6135 (5)	0.4222 (2)	0.0498 (8)	
H15A	0.8201	0.5223	0.3597	0.060*	
H15B	0.7911	0.6702	0.4150	0.060*	
C16	0.89578 (11)	0.6953 (5)	0.3813 (3)	0.0574 (9)	
H16A	0.8876	0.6275	0.3089	0.069*	
H16B	0.9106	0.6023	0.4385	0.069*	
C17	0.92853 (12)	0.8583 (6)	0.3722 (3)	0.0620 (10)	
H17A	0.9380	0.9213	0.4459	0.074*	
H17B	0.9556	0.8049	0.3508	0.074*	
C18	0.83255 (11)	0.9076 (5)	0.3240 (3)	0.0559 (9)	
H18A	0.8048	0.9589	0.3427	0.067*	
H18B	0.8242	0.8397	0.2516	0.067*	
C19	0.86433 (12)	1.0737 (5)	0.3134 (3)	0.0585 (10)	
H19A	0.8494	1.1610	0.2531	0.070*	
H19B	0.8709	1.1471	0.3841	0.070*	
C20	0.93639 (12)	1.1478 (5)	0.2585 (3)	0.0524 (8)	
C21A	0.9766 (11)	1.167 (5)	0.303 (2)	0.103 (11)	0.47 (5)
H21A	0.9892	1.0838	0.3630	0.124*	0.47 (5)
C22A	1.0052 (11)	1.307 (6)	0.269 (3)	0.184 (19)	0.47 (5)
H22A	1.0353	1.3161	0.3092	0.220*	0.47 (5)
C21B	0.9877 (9)	1.150 (3)	0.3049 (16)	0.059 (4)	0.53 (5)
H21B	1.0010	1.0609	0.3605	0.071*	0.53 (5)
C22B	1.0145 (8)	1.284 (2)	0.2650 (12)	0.066 (5)	0.53 (5)
H22B	1.0462	1.2840	0.2894	0.079*	0.53 (5)
C23	0.99198 (17)	1.4234 (8)	0.1851 (4)	0.0947 (15)	
C24	0.94645 (15)	1.4229 (6)	0.1412 (3)	0.0817 (13)	
H24	0.9337	1.5159	0.0872	0.098*	
C25	0.91882 (13)	1.2851 (6)	0.1763 (3)	0.0616 (10)	
H25	0.8876	1.2844	0.1440	0.074*	
N1	0.85421 (8)	0.7703 (4)	0.41259 (18)	0.0446 (6)	
N2	0.90743 (9)	1.0027 (4)	0.2877 (2)	0.0527 (7)	
O1	0.78550 (7)	0.3726 (3)	0.64797 (15)	0.0455 (5)	
O2	0.75946 (8)	0.5494 (4)	0.85303 (16)	0.0582 (7)	
O3	0.87276 (10)	1.0320 (4)	0.62102 (19)	0.0689 (7)	
H3	0.8708	0.9690	0.5620	0.103*	
O4	0.77554 (8)	0.2523 (4)	0.47254 (18)	0.0653 (7)	
O5A	1.0188 (7)	1.589 (4)	0.174 (3)	0.116 (8)	0.47 (5)
H5A1	1.0053	1.6567	0.1208	0.173*	0.47 (5)
O5B	1.0186 (6)	1.531 (3)	0.1318 (16)	0.080 (4)	0.53 (5)
H5B1	1.0034	1.5704	0.0709	0.120*	0.53 (5)

### Atomic displacement parameters (Å^2^)

**Table:**

	*U*^11^	*U*^22^	*U*^33^	*U*^12^	*U*^13^	*U*^23^
C1	0.0456 (18)	0.0352 (17)	0.0330 (13)	−0.0035 (15)	0.0025 (12)	−0.0011 (13)
C2	0.0496 (19)	0.0439 (19)	0.0364 (14)	−0.0019 (15)	0.0147 (13)	0.0051 (13)
C3	0.059 (2)	0.0478 (19)	0.0391 (15)	−0.0061 (18)	0.0159 (15)	−0.0022 (15)
C4	0.075 (2)	0.069 (3)	0.0551 (19)	0.004 (2)	0.0274 (18)	−0.0153 (19)
C5	0.089 (3)	0.044 (2)	0.078 (2)	0.003 (2)	0.028 (2)	−0.0179 (18)
C6	0.080 (3)	0.0266 (18)	0.0600 (19)	0.0035 (18)	0.0194 (18)	−0.0011 (15)
C7	0.068 (2)	0.040 (2)	0.0543 (18)	−0.0102 (19)	0.0107 (17)	−0.0105 (16)
C8	0.061 (2)	0.044 (2)	0.0589 (19)	−0.0102 (18)	0.0134 (16)	−0.0023 (16)
C9	0.051 (2)	0.042 (2)	0.0467 (16)	−0.0034 (16)	0.0113 (14)	−0.0057 (15)
C10	0.0417 (17)	0.0323 (17)	0.0363 (14)	−0.0004 (14)	0.0075 (12)	−0.0029 (12)
C11	0.0441 (18)	0.046 (2)	0.0355 (14)	−0.0034 (16)	0.0082 (12)	−0.0079 (14)
C12	0.050 (2)	0.0412 (19)	0.0432 (16)	0.0006 (17)	0.0074 (14)	−0.0051 (15)
C13	0.091 (3)	0.062 (2)	0.0424 (17)	−0.003 (2)	−0.0015 (17)	−0.0021 (16)
C14	0.084 (3)	0.102 (4)	0.065 (2)	−0.012 (3)	0.003 (2)	−0.029 (2)
C15	0.052 (2)	0.059 (2)	0.0403 (15)	−0.0026 (18)	0.0136 (14)	−0.0042 (15)
C16	0.056 (2)	0.055 (2)	0.068 (2)	0.0122 (19)	0.0284 (17)	0.0078 (18)
C17	0.057 (2)	0.056 (2)	0.079 (2)	0.018 (2)	0.0284 (18)	0.0184 (19)
C18	0.053 (2)	0.065 (2)	0.0532 (18)	0.0101 (19)	0.0180 (16)	0.0112 (17)
C19	0.061 (2)	0.057 (2)	0.0636 (19)	0.0196 (19)	0.0258 (17)	0.0146 (17)
C20	0.051 (2)	0.051 (2)	0.0564 (19)	0.0008 (18)	0.0149 (17)	0.0001 (17)
C21A	0.026 (12)	0.111 (16)	0.168 (17)	0.010 (9)	0.009 (8)	0.088 (13)
C22A	0.034 (10)	0.23 (3)	0.27 (3)	−0.002 (12)	−0.005 (11)	0.13 (3)
C21B	0.025 (9)	0.062 (8)	0.085 (8)	0.017 (6)	0.002 (5)	0.015 (7)
C22B	0.041 (7)	0.049 (8)	0.097 (9)	−0.011 (5)	−0.005 (5)	0.022 (6)
C23	0.072 (3)	0.087 (4)	0.116 (4)	−0.027 (3)	−0.001 (3)	0.033 (3)
C24	0.080 (3)	0.077 (3)	0.079 (2)	−0.025 (3)	−0.003 (2)	0.025 (2)
C25	0.062 (2)	0.058 (2)	0.059 (2)	−0.010 (2)	0.0006 (17)	0.0061 (19)
N1	0.0462 (16)	0.0481 (16)	0.0422 (13)	0.0059 (13)	0.0156 (11)	0.0046 (12)
N2	0.0531 (17)	0.0512 (18)	0.0590 (15)	0.0100 (15)	0.0233 (13)	0.0086 (14)
O1	0.0577 (14)	0.0379 (12)	0.0422 (11)	−0.0094 (11)	0.0136 (9)	−0.0071 (9)
O2	0.0718 (16)	0.0643 (17)	0.0440 (11)	−0.0185 (14)	0.0245 (10)	−0.0059 (11)
O3	0.100 (2)	0.0462 (15)	0.0619 (13)	−0.0122 (15)	0.0210 (14)	0.0062 (12)
O4	0.0738 (17)	0.0670 (17)	0.0549 (13)	−0.0186 (14)	0.0136 (12)	−0.0250 (12)
O5A	0.090 (7)	0.087 (11)	0.138 (16)	−0.065 (8)	−0.048 (9)	0.047 (10)
O5B	0.066 (5)	0.084 (9)	0.080 (6)	−0.054 (5)	−0.010 (5)	0.029 (6)

### Geometric parameters (Å, º)

**Table:**

C1—O1	1.454 (3)	C15—N1	1.475 (4)
C1—C2	1.490 (4)	C15—H15A	0.9700
C1—C10	1.542 (4)	C15—H15B	0.9700
C1—H1	0.9800	C16—N1	1.464 (4)
C2—O2	1.454 (3)	C16—C17	1.503 (5)
C2—C3	1.468 (4)	C16—H16A	0.9700
C2—H2	0.9800	C16—H16B	0.9700
C3—O2	1.455 (4)	C17—N2	1.459 (4)
C3—C13	1.499 (4)	C17—H17A	0.9700
C3—C4	1.499 (5)	C17—H17B	0.9700
C4—C5	1.540 (5)	C18—N1	1.462 (4)
C4—H4A	0.9700	C18—C19	1.504 (5)
C4—H4B	0.9700	C18—H18A	0.9700
C5—C6	1.505 (5)	C18—H18B	0.9700
C5—H5A	0.9700	C19—N2	1.469 (4)
C5—H5B	0.9700	C19—H19A	0.9700
C6—C7	1.314 (5)	C19—H19B	0.9700
C6—H6	0.9300	C20—C21A	1.21 (3)
C7—C14	1.516 (5)	C20—C25	1.381 (5)
C7—C8	1.516 (4)	C20—N2	1.409 (4)
C8—O3	1.426 (4)	C20—C21B	1.52 (3)
C8—C9	1.539 (5)	C21A—C22A	1.41 (4)
C8—H8	0.9800	C21A—H21A	0.9300
C9—C10	1.530 (4)	C22A—C23	1.27 (3)
C9—H9A	0.9700	C22A—H22A	0.9300
C9—H9B	0.9700	C21B—C22B	1.37 (3)
C10—C11	1.526 (4)	C21B—H21B	0.9300
C10—H10	0.9800	C22B—C23	1.415 (18)
C11—C12	1.499 (4)	C22B—H22B	0.9300
C11—C15	1.543 (4)	C23—O5B	1.340 (15)
C11—H11	0.9800	C23—C24	1.350 (6)
C12—O4	1.208 (3)	C23—O5A	1.411 (17)
C12—O1	1.347 (3)	C24—C25	1.377 (5)
C13—H13A	0.9600	C24—H24	0.9300
C13—H13B	0.9600	C25—H25	0.9300
C13—H13C	0.9600	O3—H3	0.8200
C14—H14A	0.9600	O5A—H5A1	0.8200
C14—H14B	0.9600	O5B—H5B1	0.8200
C14—H14C	0.9600		
			
O1—C1—C2	107.0 (2)	N1—C15—C11	113.0 (2)
O1—C1—C10	106.13 (18)	N1—C15—H15A	109.0
C2—C1—C10	111.3 (2)	C11—C15—H15A	109.0
O1—C1—H1	110.7	N1—C15—H15B	109.0
C2—C1—H1	110.7	C11—C15—H15B	109.0
C10—C1—H1	110.7	H15A—C15—H15B	107.8
O2—C2—C3	59.74 (17)	N1—C16—C17	111.1 (3)
O2—C2—C1	120.0 (3)	N1—C16—H16A	109.4
C3—C2—C1	126.1 (3)	C17—C16—H16A	109.4
O2—C2—H2	113.5	N1—C16—H16B	109.4
C3—C2—H2	113.5	C17—C16—H16B	109.4
C1—C2—H2	113.5	H16A—C16—H16B	108.0
O2—C3—C2	59.63 (18)	N2—C17—C16	111.2 (3)
O2—C3—C13	112.6 (3)	N2—C17—H17A	109.4
C2—C3—C13	122.0 (3)	C16—C17—H17A	109.4
O2—C3—C4	116.5 (3)	N2—C17—H17B	109.4
C2—C3—C4	116.6 (3)	C16—C17—H17B	109.4
C13—C3—C4	116.5 (3)	H17A—C17—H17B	108.0
C3—C4—C5	111.8 (3)	N1—C18—C19	111.0 (3)
C3—C4—H4A	109.3	N1—C18—H18A	109.4
C5—C4—H4A	109.3	C19—C18—H18A	109.4
C3—C4—H4B	109.3	N1—C18—H18B	109.4
C5—C4—H4B	109.3	C19—C18—H18B	109.4
H4A—C4—H4B	107.9	H18A—C18—H18B	108.0
C6—C5—C4	111.4 (3)	N2—C19—C18	111.5 (3)
C6—C5—H5A	109.4	N2—C19—H19A	109.3
C4—C5—H5A	109.4	C18—C19—H19A	109.3
C6—C5—H5B	109.4	N2—C19—H19B	109.3
C4—C5—H5B	109.4	C18—C19—H19B	109.3
H5A—C5—H5B	108.0	H19A—C19—H19B	108.0
C7—C6—C5	128.8 (3)	C21A—C20—C25	115.6 (14)
C7—C6—H6	115.6	C21A—C20—N2	124.6 (14)
C5—C6—H6	115.6	C25—C20—N2	119.8 (3)
C6—C7—C14	126.0 (3)	C21A—C20—C21B	7 (2)
C6—C7—C8	121.7 (3)	C25—C20—C21B	117.1 (10)
C14—C7—C8	112.2 (3)	N2—C20—C21B	122.9 (10)
O3—C8—C7	111.4 (3)	C20—C21A—C22A	123 (2)
O3—C8—C9	111.2 (3)	C20—C21A—H21A	118.3
C7—C8—C9	110.7 (3)	C22A—C21A—H21A	118.3
O3—C8—H8	107.8	C23—C22A—C21A	124 (2)
C7—C8—H8	107.8	C23—C22A—H22A	118.2
C9—C8—H8	107.8	C21A—C22A—H22A	118.2
C10—C9—C8	116.9 (3)	C22B—C21B—C20	120.0 (17)
C10—C9—H9A	108.1	C22B—C21B—H21B	120.0
C8—C9—H9A	108.1	C20—C21B—H21B	120.0
C10—C9—H9B	108.1	C21B—C22B—C23	117.2 (17)
C8—C9—H9B	108.1	C21B—C22B—H22B	121.4
H9A—C9—H9B	107.3	C23—C22B—H22B	121.4
C11—C10—C9	114.5 (2)	C22A—C23—O5B	127.0 (17)
C11—C10—C1	102.9 (2)	C22A—C23—C24	115.0 (15)
C9—C10—C1	115.9 (2)	O5B—C23—C24	117.8 (8)
C11—C10—H10	107.7	C22A—C23—O5A	119.3 (17)
C9—C10—H10	107.7	O5B—C23—O5A	26.9 (13)
C1—C10—H10	107.7	C24—C23—O5A	120.6 (10)
C12—C11—C10	104.8 (2)	C22A—C23—C22B	13 (2)
C12—C11—C15	109.2 (2)	O5B—C23—C22B	116.5 (12)
C10—C11—C15	113.6 (3)	C24—C23—C22B	123.9 (9)
C12—C11—H11	109.7	O5A—C23—C22B	114.0 (11)
C10—C11—H11	109.7	C23—C24—C25	120.2 (4)
C15—C11—H11	109.7	C23—C24—H24	119.9
O4—C12—O1	120.6 (3)	C25—C24—H24	119.9
O4—C12—C11	128.4 (3)	C24—C25—C20	121.5 (4)
O1—C12—C11	110.9 (2)	C24—C25—H25	119.3
C3—C13—H13A	109.5	C20—C25—H25	119.3
C3—C13—H13B	109.5	C18—N1—C16	107.9 (2)
H13A—C13—H13B	109.5	C18—N1—C15	109.0 (2)
C3—C13—H13C	109.5	C16—N1—C15	112.3 (3)
H13A—C13—H13C	109.5	C20—N2—C17	116.9 (3)
H13B—C13—H13C	109.5	C20—N2—C19	115.6 (3)
C7—C14—H14A	109.5	C17—N2—C19	110.2 (2)
C7—C14—H14B	109.5	C12—O1—C1	110.8 (2)
H14A—C14—H14B	109.5	C2—O2—C3	60.63 (18)
C7—C14—H14C	109.5	C8—O3—H3	109.5
H14A—C14—H14C	109.5	C23—O5A—H5A1	109.5
H14B—C14—H14C	109.5	C23—O5B—H5B1	109.5

### Hydrogen-bond geometry (Å, º)

**Table:**

*D*—H···*A*	*D*—H	H···*A*	*D*···*A*	*D*—H···*A*
O3—H3···N1	0.82	2.22	3.030 (4)	169
C2—H2···O4^i^	0.98	2.45	3.243 (4)	138
C4—H4*A*···O2^ii^	0.97	2.43	3.315 (5)	151

Symmetry codes: (i) −*x*+3/2, *y*+1/2, −*z*+1; (ii) −*x*+3/2, *y*+1/2, −*z*+2.

## References

[bb1] Abdel Sattar, E., Galal, A. M. & Mossa, J. S. (1996). *J. Nat. Prod.* **59**, 403–405. 10.1021/np960064g 8699183

[bb2] Bruker (2009). *APEX2*, *SAINT* and *SADABS* Bruker AXS Inc., Madison, Wisconsin, USA.

[bb3] Cremer, D. & Pople, J. A. (1975). *J. Am. Chem. Soc.* **97**, 1354–1358.

[bb4] El Hassany, B., El Hanbali, F., Akssira, M., Mellouki, F., Haidou, A. & Barero, A. F. (2004). *Fitoterapia*, **75**, 573–576. 10.1016/j.fitote.2004.06.003 15351111

[bb5] Farrugia, L. J. (2012). *J. Appl. Cryst.* **45**, 849–854.

[bb6] Hwang, D.-R., Wu, Y.-S., Chang, C.-W., Lien, T.-W., Chen, W.-C., Tan, U.-K., Hsu, J. T. A. & Hsieh, H.-P. (2006). *Bioorg. Med. Chem.* **14**, 83–91. 10.1016/j.bmc.2005.07.055 16140536

[bb7] Moumou, M., Benharref, A., Daran, J.-C., Mellouki, F. & Berraho, M. (2012). *Acta Cryst.* E**68**, o589–o590. 10.1107/S1600536812003662 PMC3297315 22412505

[bb8] Neelakantan, S., Nasim, Sh., Guzman, M. L., Jordan, C. T. & Crooks, P. A. (2009). *Bioorg. Med. Chem. Lett.* **19**, 4346–4349. 10.1016/j.bmcl.2009.05.092 19505822

[bb9] Sheldrick, G. M. (2008). *Acta Cryst.* A**64**, 112–122. 10.1107/S0108767307043930 18156677

[bb10] Spek, A. L. (2009). *Acta Cryst.* D**65**, 148–155. 10.1107/S090744490804362X PMC2631630 19171970

[bb11] Westrip, S. P. (2010). *J. Appl. Cryst.* **43**, 920–925.

